# The Development of a Yolov8-Based Model for the Measurement of Critical Shoulder Angle (CSA), Lateral Acromion Angle (LAA), and Acromion Index (AI) from Shoulder X-ray Images

**DOI:** 10.3390/diagnostics14182092

**Published:** 2024-09-22

**Authors:** Turab Selçuk

**Affiliations:** Department of Electrical and Electronics Engineering, Kahramanmaras Sutcu Imam University, 46050 Onikişubat, Turkey; turabselcuk23@ksu.edu.tr

**Keywords:** shoulder X-ray images, Yolov8, instance segmentation

## Abstract

**Background:** The accurate and effective evaluation of parameters such as critical shoulder angle, lateral acromion angle, and acromion index from shoulder X-ray images is crucial for identifying pathological changes and assessing disease risk in the shoulder joint. **Methods:** In this study, a YOLOv8-based model was developed to automatically measure these three parameters together, contributing to the existing literature. Initially, YOLOv8 was used to segment the acromion, glenoid, and humerus regions, after which the CSA, LAA angles, and AI between these regions were calculated. The MURA dataset was employed in this study. **Results:** Segmentation performance was evaluated with the Dice and Jaccard similarity indices, both exceeding 0.9. Statistical analyses of the measurement performance, including Pearson correlation coefficient, RMSE, and ICC values demonstrated that the proposed model exhibits high consistency and similarity with manual measurements. **Conclusions:** The results indicate that automatic measurement methods align with manual measurements with high accuracy and offer an effective alternative for clinical applications. This study provides valuable insights for the early diagnosis and management of shoulder diseases and makes a significant contribution to existing measurement methods.

## 1. Introduction

The shoulder joint is very mobile, exhibiting a wide range of motion. The extensive degree of mobility places considerable strain on the adjacent structures and increases susceptibility to numerous diseases. Shoulder diseases, specifically rotator cuff tears (RCT) and glenohumeral osteoarthritis, are commonly seen issues. The anatomical structure of the shoulder is a key factor in the development of these disorders [1,2].

In the anatomical assessment of the shoulder joint, radiographic measurements play a crucial role. Among these measurements, critical shoulder angle (CSA), lateral acromion angle (LAA), and acromion index (AI) emerge as critical parameters associated with shoulder pathologies [3,4]. CSA is an angle that affects the biomechanics of the shoulder joint and is directly related to the risk of RCT and osteoarthritis [5,6]. The LAA influences shoulder stability and load distribution by determining the inclination of the acromion. Similarly, AI is a parameter that can be evaluated to determine the likelihood of RCT [7]. The calculation of these parameters requires careful and time-consuming examination. This increases the need for artificial intelligence based diagnostic systems, which have the potential to optimize diagnostic processes by providing faster and more accurate results to orthopedists. Consequently, deep learning-based research has gained momentum in recent times, and these technologies have been extensively used for the automatic analysis of medical images, such as shoulder radiographs. For instance, deep neural networks have been utilized on magnetic resonance images (MRI) to classify RCT [8] and to segment both rotator cuff muscles [9,10] and the glenohumeral joint [11]. Additionally, deep learning has been used to quantify and characterize rotator cuff muscle degeneration from CT scans [12]. In the context of radiographs, deep learning techniques have been employed to detect humerus fractures [13] and to classify shoulder implants [14]. Moreover, artificial intelligence has been applied for anatomic region detection in radiographic images to measure the CSA, providing accurate, reproducible, and rapid measurements [15].

In this study, a YOLOv8-based model was developed to enable orthopedic specialists to rapidly and comprehensively evaluate angles and indices in shoulder X-rays, which are typically time-consuming and demanding. The YOLOv8 model outperforms other models, particularly in terms of speed and high accuracy. Additionally, it successfully segments complex anatomical structures in medical images, such as shoulder X-rays. The uniqueness of this study lies in the simultaneous calculation of the CSA, AI, LAA for a comprehensive analysis of shoulder X-ray images. To achieve this, YOLOv8-based segmentation models and mathematical morphology techniques were employed to accurately segment the acromion, humerus, and glenoid from shoulder X-ray images. The developed model aims to provide an effective tool for the early diagnosis and treatment planning of shoulder pathologies. The combined evaluation of CSA, AI, and LAA offers a more comprehensive understanding of shoulder joint biomechanics and pathologies, contributing to accurate diagnosis and the development of personalized treatment strategies. The study addressed the challenges of manual measurements in clinical practice by offering an automated solution and emphasized the importance of considering these critical parameters together.

## 2. Materials and Methods

**Figure 1:**
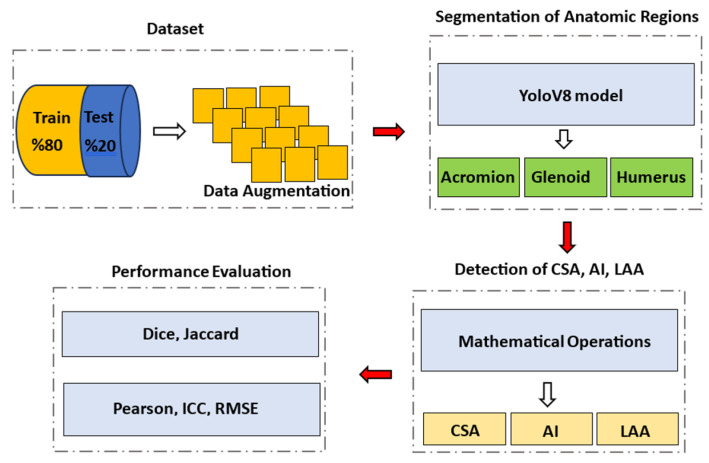
The flowchart of the methods.

The detection of CSA, AI, and LAA from X-ray images was achieved through four stages. The first stage involved the creation of the dataset, during which shoulder X-ray images were collected, and three anatomical regions—the acromion, humerus, and glenoid—were labeled within these images. The second stage involved the development of a YOLOv8-based segmentation model to automatically detect these anatomical regions. The third stage included performing pixel-based mathematical operations to calculate CSA, AI, and LAA. In the final stage, the performance of YOLOv8 was tested in comparison with U-Net using the Dice and Jaccard similarity indices, along with statistical analysis. The flowchart summarizing the methods used is shown in Figure 1.

### 2.1. Materials

In this study, the MURA public dataset was used. The MURA dataset is a comprehensive collection of musculoskeletal images, consisting of 14,863 studies from 12,173 patients and including a total of 40,561 multiview radiographs [16,17]. The dataset provides images from seven standard upper extremity radiography types: elbow, finger, forearm, hand, humerus, shoulder, and wrist. For the calculation of CSA, AI, and LAA together, 217 high-quality images were selected, in which the anatomical structures of the acromion, glenoid, and humerus were clearly visible. The selection process was guided by the criterion that these structures must be accurately measurable, ensuring that the images were high-resolution and of good quality. Additionally, data augmentation techniques were applied to increase the diversity of the dataset and improve the model’s performance and generalization ability. A portion of the dataset was labeled in DICOM format by six Stanford-approved radiologists, and these labels were used to evaluate the model’s performance. The average experience of the radiologists was 8.83 years, with a range between 2 and 25 years [18].

### 2.2. Anatomical Structure Segmentation (YOLOv8)

To calculate CSA, AI, and LAA from shoulder X-ray images, it is essential to accurately delineate the boundaries of the acromion, glenoid, and humerus. In this study, a YOLOv8-based [19] segmentation model was developed. The YOLOv8-seg model adopts the YOLACT [20] approach, dividing object segmentation into two parallel processes: object detection and instance segmentation. While object detection identifies individual objects within the image, instance segmentation analyzes the meaning of each pixel to distinguish object classes and provide context. The results of these two processes were combined to create precise masks that accurately defined the boundaries of the objects.

The YOLOv8-seg architecture also includes an output module specifically designed to generate mask coefficients and FCN layers with a Proto module that plays a crucial role in mask production. The Proto module creates and refines masks through convolutional operations. Additionally, a specialized loss term for segmentation is included. This integrated structure allows the model to segment objects in medical images accurately and efficiently, providing valuable insights.

The YOLOv8 model uses a loss function composed of three main components. These components are: the bounding box *Loss* ($L_{box}$), which measures the difference between the predicted and the true bounding boxes; objectness loss, which predicts whether an object exists within a cell; and segmentation loss ($L_{seg}$), which evaluates whether the boundaries of the detected object are correctly delineated, along with classification loss ($L_{cls}$) which assesses whether the detected object is classified correctly. This loss function can be generally formulated as shown in Equation (1):

(1) $$Loss=L_{box}+L_{seg}+L_{cls}$$

$L_{cls}$ utilizes a variant of cross-entropy loss, specifically binary cross-entropy (BCE) loss, as shown in Equation (2). In this, W represents the weight, $x_{n}$ indicates the predicted class value, and $y_{n}$ refers to the true labeled value. This loss measures the error associated with object classification, capturing the difference between the predicted and actual class labels.

(2) $$L_{cls}=-W[y_{n}\log\left(x_{n}\right)+1-y_{n}\log\left(1-x_{n}\right)]$$

$L_{seg}$ is specifically designed for segmentation tasks. It assesses the difference between the instance segmentation produced by the model and the corresponding ground truth segmentation. $L_{box}\;$computes the loss using the complete intersection over union (CIoU) loss [21]. This metric measures the difference between the predicted bounding box tuple (*x*, *y*, width, height) and the actual ground truth tuple that encapsulates the object. CIoU loss takes into account the overlap between the two boxes as well as the differences in their aspect ratios. Equation (3) defines the *CIoU* loss.

(3) $$L_{CIoU}=1-IoU+\frac{\rho^{2}\left(b,b^{gt}\right)}{c^{2}}+\alpha v$$

(4) $$\alpha =\frac{v}{(1-IoU)+v}$$

(5) $$v=\frac{4}{\pi^{2}}{\left(arctan\frac{w^{gt}}{h^{gt}}-arctan\frac{w}{h}\right)}^{2}$$

In this context, b and $b^{gt}$ represent the central points of the predicted bounding box and the ground truth box, respectively. The parameter is a hyperparameter, while v measures the consistency of the aspect ratio, as defined by Equations (4) and (5), respectively. The Euclidean distance is represented by ρ, and c denotes the diagonal length of the smallest enclosing box that contains both bounding boxes.

### 2.3. Radiographic Measurements

**Figure 2:**
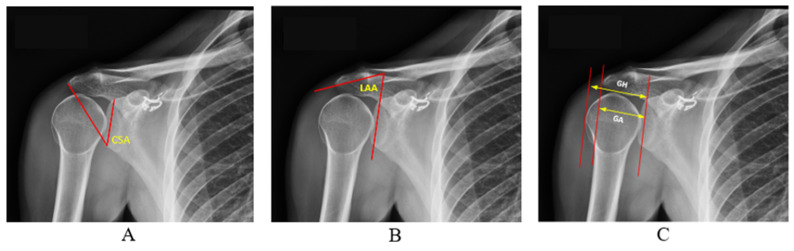
Representation of CSA (**A**), LAA (**B**), and AI (**C**).

As shown in Figure 2, three different radiographic measurements were derived using these boundary pixels: *CSA*, *AI*, and *LAA*.

At this stage, the edge pixels of the segmented glenoid, acromion, and humerus were obtained. Using these edge pixels, the measurements for CSA, AI, and LAA were calculated. For the CSA measurement, we first defined the upper part of the glenoid (G) as the point where the upper edge of the glenoid fossa intersects with a vertical line. Similarly, the lower part of the glenoid (I) is defined as the point where the lower edge intersects with the same vertical line. The lateral edge of the acromion (A) is the outermost point of the acromion. Following these definitions, a vertical line is drawn from the upper part to the lower part of the glenoid (G-I line). Let the slope of this line be $m_{1}$. Then, another line is drawn from the lower part of the glenoid (I) to the lateral edge of the acromion. Let the slope of this line be $m_{2}$. The CSA is the angle between these two lines, mathematically expressed as in Equation (6).

(6) $$CSA=\tan^{-1}\frac{m_{1}-m_{2}}{1+m_{1}m_{2}}$$

AI is defined as the ratio of the distance between the glenoid fossa and the lateral edge of the acromion (GA) to the distance between the glenoid edge and the lateral surface of the humeral head (GH). Its mathematical expression is given in Equation (7)

(7) $$\;AI=\frac{GA}{GH}$$

Let the slope of the line GA, drawn between a point on the glenoid cavity and a point on the lateral edge of the acromion, be $n_{1}$, and the slope of the line GH, drawn from the glenoid cavity to the head of the humerus, be $n_{2}$. The lateral acromial angle is the angle formed by the intersection of these two lines. Its mathematical expression is given in Equation (8).

(8) $$\;LAA=\tan^{-1}\frac{n_{1}-n_{2}}{1+n_{1}n_{2}}$$

### 2.4. Performance Metrics

Performance metrics are crucial for measuring the accuracy and effectiveness of machine learning and image processing algorithms. To evaluate the segmentation performance of anatomical regions, the Dice similarity coefficient (DSC) [22] and Jaccard [23] index were used and can be calculated as shown in Equations (9) and (10).

(9) $$DSC=2\times \frac{A\cap B}{\left|A\right|+|B|}$$

(10) $$Jaccard=\frac{A\cap B}{A\cup B}$$

Here, *A* represents the automatically detected area, while *B* represents the manually detected area. The manual detection was performed by an orthopedic specialist with five years of experience.

### 2.5. Statistical Analysis

The strength of the relationships between the automatic and manual measurements was evaluated in the study using both the Pearson correlation coefficient and the intraclass correlation coefficient via the one-way random effects model (ICC (1,1)). To assess the reliability and consistency of the measurements, both manual and automatic procedures were repeated three times. The ICC and root mean square error (RMSE) values derived from these repetitions are as follows. The gold standard for comparison was the manual measurements performed by an orthopedist with five years of experience. The ICC values were interpreted according to the following classification: ≥0.9 as excellent, 0.75 to 0.89 as good, 0.5 to 0.74 as moderate, and <0.49 as indicating poor reliability. The RMSE between the different measurements was also calculated, which quantifies the deviation of the predicted values from the actual measured values based on Euclidean distance. RMSE was calculated by first determining the residual value, or the difference between the predicted and actual measurements, for each data point. This was followed by averaging these residuals and taking the square root of the mean.

## 3. Results and Discussion

**Figure 3:**
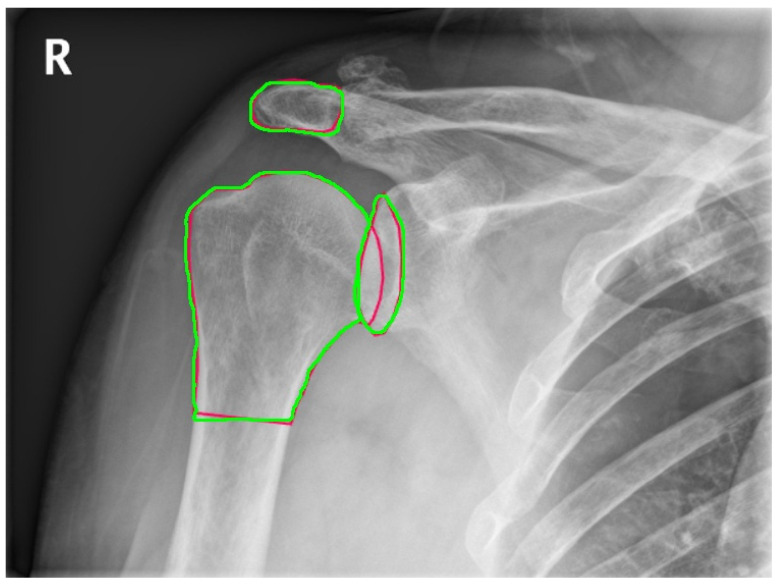
The anatomic regions detected automatically (red) and manually(green).

In the study, 217 shoulder X-ray images from the MURA dataset were used. Out of these images, 140 were allocated for training and 77 for testing. Data augmentation, including rotations and symmetries along the x and y axes, increased the number of training images to 560. The YOLOv8 model was trained with this dataset over 100 epochs, using a learning rate of 0.01 and the Adam optimizer. Figure 3 shows the regions detected automatically and manually. The green drawing represents manual detection by the orthopedic specialist, while the red drawing represents detection by YOLOv8. As shown in Figure 3 a difference was observed between manual and automatic methods in delineating the boundaries of the lower sections of the humerus. This difference arises from the data labeling process, where careful labeling was not performed, rather than affecting the angle calculations of this lower section. The model demonstrates high performance in delineating the boundaries of the glenoid and acromion.

The segmentation performance of YOLOv8 and U-Net was evaluated using the Dice similarity coefficient (DSC) and Jaccard indices for the acromion, glenoid, and humerus regions, with the results shown in Table 1. For the acromion region, YOLOv8 achieved a DSC value of 0.91 and a Jaccard index of 0.90, indicating high accuracy in acromion segmentation, though slightly lower performance compared to other regions. For the glenoid region, the DSC was 0.93 and the Jaccard index was 0.92, demonstrating successful glenoid segmentation as well. The highest performance was observed in the humerus region, with a DSC value of 0.97 and a Jaccard index of 0.96, indicating that YOLOv8 provided excellent accuracy in humerus segmentation. Overall, YOLOv8’s segmentation performance was high, with the best results seen in the humerus region, likely due to its more distinct structure compared to the other two regions.

In comparison, for the acromion region, U-Net achieved a DSC value of 0.79 and a Jaccard index of 0.77, which shows lower accuracy in acromion segmentation compared to YOLOv8. In the glenoid region, U-Net obtained a DSC value of 0.74 and a Jaccard index of 0.71, indicating lower segmentation accuracy compared to YOLOv8. For the humerus region, U-Net achieved a DSC value of 0.94 and a Jaccard index of 0.92, demonstrating better performance in humerus segmentation, but still lower compared to YOLOv8’s superior performance. Overall, U-Net’s segmentation results showed significantly lower index values and a clear performance gap compared to YOLOv8.

**Figure 4:**
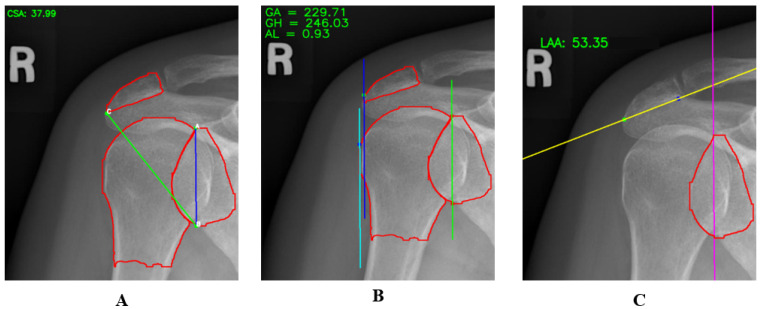
Example images showing the measured values of CSA (**A**), AI (**B**), and LAA (**C**).

Table 2 shows the distribution of CSA, LAA, and AI values among individuals, which is important for understanding the structural features potentially associated with shoulder pathologies. Individuals with CSA values below 30 generally exhibit a lower risk of disease, while those with values between 30 and 35 degrees may have a moderate risk. CSA values above 35 may indicate a high risk and are often associated with issues such as shoulder impingement syndrome and rotator cuff tendinitis [24]. Individuals with an LAA value less than 45 degrees generally have a moderate to low risk, while values between 45 and 55 degrees are considered normal. LAA values above 55 are associated with a high-risk group and are commonly linked to rotator cuff pathologies [25]. AI values reflect acromion width; individuals with AI < 0.7 typically have a moderate to high risk, which may indicate pathological effects of a narrow acromion. Those with AI values between 0.7 and 1 have a normal risk level, while individuals with AI ≥ 1 have a wider acromion, which may increase the risk of shoulder pathologies [26]. Considering these values in clinical assessments is crucial for better understanding and managing potential issues in the shoulder joint. Table 3 also shows the mean, standard deviation, minimum, and maximum values for CSA, AI, and LAA. Figure 4 shows the measured CSA, AI, and LAA values on example images. The segmented acromion, glenoid, and humerus are shown in red. AI is a distance, while CSA and LAA are angles.

To evaluate the reliability of the measurements, both manual and automatic measurements were repeated three times. The measurement results are shown in Table 4. The intraclass correlation coefficient (ICC) was calculated for these measurements using a one-way random effects model (ICC(1,1)). For CSA, the ICC value for manual assessment was found to be 0.89 (95% CI: 0.85–0.92), while for automatic assessment it was 0.91 (95% CI: 0.88–0.95). For the AI, the manual ICC was 0.91 (95% CI: 0.88–0.94), and the automatic ICC was 0.92 (95% CI: 0.89–0.96). For LAA, the manual ICC was 0.90 (95% CI: 0.87–0.93), and the automatic ICC was 0.92 (95% CI: 0.88–0.95).

The deviations in manual measurements are directly related to the accurate identification of the reference points for the glenoid and acromion, as well as the inclination of the lateral edge of the acromion. In each measurement, the reference points are marked with a variation of one or two pixels, causing minor deviations in the measurements. However, the high ICC values indicated that the manual measurements were consistent and reliable.

The ICC values obtained from automatic measurements using YOLOv8 also demonstrated that the measurements were consistent and reliable. The RMSE values in automatic measurements were lower compared to manual measurements, indicating less deviation in automatic measurements. This was an expected outcome, as visual errors may occur in manual measurements due to the expert’s workload. The deviations in automatic measurements were associated with the training parameters or the quality of the training dataset.

Table 5 presents the statistical analysis between manual and automatic measurements. Pearson correlation values, with 0.97 for CSA, 0.98 for AI, and 0.95 for LAA, indicate a very strong linear relationship between manual and automatic measurements. Additionally, the ICC (intraclass correlation coefficient) values were measured at high levels, with 0.94 for CSA, 0.98 for AI, and 0.94 for LAA, confirming that the agreement between manual and automatic measurements was excellent and that the measurement reliability was high.

The RMSE values indicated the average deviations between manual and automatic measurements, and the RMSE values for CSA, AI, and LAA were measured as 2.89, 0.38, and 1.96, respectively. The higher RMSE value for CSA reflects the challenges arising from the inclined and complex structure of the acromion and glenoid, while the lower RMSE value for AI was due to the more distinct structure of the humerus. As seen in Table 1, the performance in segmenting the humerus was higher compared to that of the acromion and glenoid. The RMSE values between automatic and manual measurements were similar to observed RMSE values in repeated manual measurements. Repeated automatic measurements have also demonstrated greater consistency. This indicates that the differences between automatic and manual measurements were actually due to clinical conditions that can also occur during repeated manual measurements. Consequently, the proposed model will enable expert clinicians to quickly and accurately compute the relevant angles from shoulder X-rays in practice.

One of the limitations of the study is that the measurements were conducted by a single evaluator. To mitigate this limitation, repeated measurements were performed by the same evaluator. Another limitation is that image preprocessing techniques were not used for images where even experts found it challenging to delineate the acromion and glenoid boundaries. Applying image preprocessing techniques to such images could enhance the model’s performance and broaden its applicability.

## 4. Conclusions

This study evaluated the performance of a YOLOv8-based deep learning model developed to automatically detect the CSA angle, acromion index, and LAA angle from shoulder X-ray images. The model’s performance was validated via statistical analyses by comparing it with manual measurements. The findings revealed a high correlation between automatic and manual measurements, indicating that the YOLOv8 model can reliably detect key angles in shoulder X-ray images and holds potential for clinical applications. In a clinical setting, such automatic systems could accelerate the measurement process, reduce human error, and provide more consistent and accurate results. This study makes a significant contribution to the literature as the first to simultaneously assess CSA, acromion index, and LAA. Future studies plan to modify the model to detect different angles and anatomical regions in shoulder radiographs, thereby broadening the scope of AI-based models in shoulder X-ray evaluations. In conclusion, such AI-supported systems can deliver rapid and effective results in busy clinical environments, providing significant advantages for clinicians in terms of time management and decision-making processes.

## Figures and Tables

**Table 1:** Comparison of YOLOv8’s segmentation performance of anatomical regions with U-Net.

	YoloV8		U-Net	
	DSC	JACCARD	DSC	JACCARD
Acromion	0.91	0.90	0.79	0.77
Glenoid	0.93	0.92	0.74	0.71
Humerus	0.97	0.96	0.94	0.92

**Table 2:** Distribution of automatically detected CSA, LAA, and AI values among individuals.

Measurement	Range	Number of Individuals
CSA	<30	24
	30 ≤ CSA < 35	21
	≥35	32
LAA	<45	13
	45 ≤ LAA ≤ 55	37
	>55	27
AI	<0.7	22
	0.7 ≤ AI < 1	29
	≥1	26

**Table 3:** Statistics of manual and automatic measurements.

		CSA	AI	LAA
MANUAL	mean(std)	36.9 (4.63)	0.75 (0.11)	52.5 (6.87)
	min-max	24.3–50.1	0.44–0.98	32.7–72.9
AUTOMATIC	mean(std)	34.2 (4.12)	0.70 (0.21)	54 (5.95)
	min-max	21.3–48.3	0.43–0.95	31.8–74.9

**Table 4:** The ICC and RMSE values for manual and automatic measurements were repeated three times.

Measurement	Evaluator	ICC (95% CI)	RMSE
CSA	Manual	0.89 (0.85–0.92)	2.16
CSA	AI	0.91 (0.88–0.95)	1.98
AI	Manual	0.91 (0.88–0.94)	0.26
AI	AI	0.92 (0.89–0.96)	0.19
LAA	Manual	0.90 (0.87–0.93)	1.33
LAA	AI	0.92 (0.88–0.95)	1.14

**Table 5:** Statistical analysis of manual and YOLOv8 measurements.

	PEARSON r(P)	ICC (95% CI)	RMSE
CSA	0.97 (<0.05)	0.94 (0.90–0.97)	2.89
AI	0.98 (<0.05)	0.98 (0.96–0.99)	0.38
LAA	0.95 (<0.05)	0.94 (0.89–0.96)	1.96

## Data Availability

The original contributions presented in the study are included in the article, further inquiries can be directed to the corresponding author.
